# Increased plasma levels of erythropoietin after renal denervation in patients with resistant hypertension

**DOI:** 10.1038/s41440-025-02317-6

**Published:** 2025-08-14

**Authors:** Jianzhong Xu, Gianni Sesa-Ashton, Nina Eikelis, Antony Walton, Elisabeth A. Lambert, Murray D. Esler, Marcio G. Kiuchi, Revathy Carnagarin, Gavin W. Lambert, Markus P. Schlaich

**Affiliations:** 1 Neurovascular Hypertension & Kidney Disease Lab/Human Neurotransmitter Lab, Baker Heart & Diabetes Institute, Melbourne, VIC Australia; 2 Department of Hypertension, Ruijin Hospital, Shanghai Jiaotong University School of Medicine, Shanghai, China; 3 Heart Centre Alfred Hospital, Melbourne, VIC Australia; 4 Iverson Health Innovation Research Institute and Faculty of Health, Arts and Design, Swinburne University of Technology, Melbourne, VIC Australia; 5 Dobney Hypertension Centre, Medical School - Royal Perth Hospital / RPH Medical Research Foundation, University of Western Australia, Perth, WA Australia

**Keywords:** Renal denervation, Sympathetic nervous system, Erythropoietin, Resistant hypertension, Blood pressure

## Abstract

Erythropoietin (Epo) plays a crucial role in the formation and maturation of erythrocytes and is produced primarily by peritubular cells in the renal cortex. Previous studies suggest that renal sympathetic nerve activity (RSNA) may influence Epo regulation. Catheter-based renal denervation (RDN) has been shown to reduce renal noradrenaline spillover and blood pressure in patients with resistant hypertension. We therefore aimed to investigate whether RDN influences Epo levels. 33 patients with resistant hypertension (age 61 ± 12 y; baseline office blood pressure (BP) 165 ± 16/85 ± 18 mmHg despite treatment with an average of 4.9 ± 1.7 antihypertensive medications) underwent bilateral RDN. Plasma levels of Epo, office blood pressure, 24 h ambulatory blood pressure monitoring (ABPM), muscle sympathetic nerve activity (MSNA), renal function and haemoglobin were measured before the procedure and at 3 months follow-up. Antihypertensive medication was not changed during the first 3 months after RDN. Office blood pressure was reduced by 15 ± 19/5 ± 12 mmHg at 3 months after denervation (*p* < 0.001 for systolic BP; *p* = 0.033 for diastolic BP). 24h-mean systolic and diastolic BP were also reduced (from 145 ± 16 to 140 ± 18 mmHg; *p* = 0.036, and from 80 ± 11 to 77 ± 12 mmHg; *p* = 0.024), respectively. While haemoglobin levels remained unchanged, a significant increase in plasma Epo levels was observed at 3 months after RDN (7.81 ± 3.68 versus 9.88 ± 5.06 mIU/mL; *p* = 0.025). Changes in Epo levels correlated with baseline MSNA (*r* = 0.580, *p* = 0.004 for burst frequency; *r* = 0.471, *p* = 0.023 for burst incidence), such that the increase in Epo was most pronounced in patients with high baseline MSNA. The RDN-induced changes in MSNA tended to correlate inversely with changes in Epo levels (*r* = −0.402; *p* = 0.064). Our findings suggest that RDN is associated with increased plasma levels of Epo in the absence of changes in haemoglobin, and that these effects are possibly mediated via a reduction in sympathetic nerve activity.

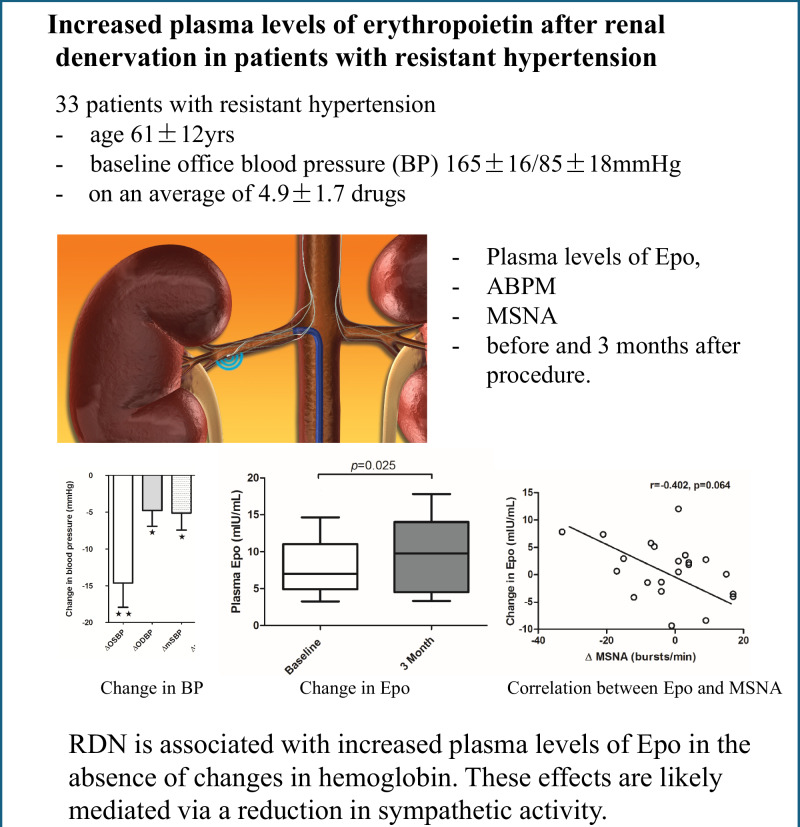

## Introduction

Erythropoietin (Epo) is a glycoprotein hormone that plays a crucial role in the formation and maturation of erythrocytes, and the major site of Epo production is the peritubular fibroblasts located within the renal cortex [1, 2]. It is generally accepted that Epo levels increase following an anaemic or hypoxaemic stimulus [3]. The kidney is particularly sensitive to both hypoxia and hypovolemia, and is thus uniquely situated to sense when an increase in oxygen-carrying capacity is required. The loss of Epo production, downstream of pericyte transdifferentiation to non-Epo producing myofibroblasts, provokes significant anaemia in patients with chronic kidney disease [4]. Renal sympathetic innervation, and renal sympathetic nerve activity itself, are considered important extrarenal modulators of Epo production [5–8]. Mechanistically, it is poorly understood exactly how interactions between sympathetic fibres and peritubular fibroblasts occur. Regardless, sympathetic control appears to be a clinically relevant modulator of Epo production, given the observation that anaemia in primary autonomic failure is independent of haematinic concentrations and is corrected by Epo supplementation [9]. Indeed, in the acute phase following haemorrhage, in which RSNA paradoxically falls, there is increased transcription and production of Epo [5].

Earlier studies investigating the role of renal nerves have shown conflicting results. Beynon demonstrated that the serum level of Epo of hypoxic rats was increased by section of the renal nerves [10]. Gebhard et al. reported that renal denervation (RDN) increased Epo secretion stimulated by carbon monoxide in rats [11]. A recent study showed Epo was increased after 120 min of transient hypotensive haemorrhage in RDN rats in normoxic conditions [5]. Others had contrasting results. Fink et al found that Epo levels were markedly reduced by prior surgical denervation of both kidneys within 5 h [12]. In a recent report, unilaterally denervated rats were exposed to different hypoxic stimuli, but no differences in Epo mRNA levels were found when innervated and denervated kidneys were compared [13]. As such, there remains significant conflict in the literature around the role of renal sympathetic innervation and Epo production.

Recent clinical trials have continued to illustrate that catheter-based RDN, the recently FDA-approved and ESH 2023 guideline-endorsed interventional approach to target both efferent sympathetic and afferent sensory nerve traffic, is a safe and effective procedure, leading to clinically relevant blood pressure reductions in patients with resistant hypertension [14–16]. A recent study from our group found a potential increase of haemoglobin concentration after RDN in patients with stage 3–4 CKD [17]. Given that Epo stimulates red blood cell production and maturation, and itself is under renal control, the question remains whether the interruption of sympathetic nerve traffic promotes this increase in haemoglobin.

This study, therefore, aimed to assess whether RDN impacts the renal synthesis of Epo in patients with resistant hypertension.

## Methods

### Study subjects

Thirty-three resistant hypertensive patients (7 female and 26 male) were enroled in our therapeutic RDN programme as extensions to the Symplicity HTN-2 trial (www.clinicaltrials.gov, NCT00888433). Eighteen patients were included in the Symplicity HTN-2 trial in Melbourne, Australia [14]. Enrolment criteria were the same as Symplicity HTN-2 study. Patients had to be above 18 years of age, had an office systolic BP of ≥160 mmHg (≥150 mmHg for patients with type 2 diabetes mellitus) despite being treated with at least three antihypertensive drugs (including one diuretic), with no changes in medication for a minimum of 2 weeks before enrolment. Patients underwent a complete medical history and physical examination. Hypertension was diagnosed based on the contemporary European Society of Hypertension and European Society of Cardiology guidelines for the management of arterial hypertension [18]. The study was approved by the Institutional Ethics Committee, and written informed consent was obtained from all patients. All patients were studied at baseline and 3-month follow-up.

### Laboratory analyses

At baseline, blood was sampled during the day with subjects in the sitting position after resting in the clinic. Thereafter, blood was centrifuged and stored at −80 °C until analysis. Plasma Epo concentrations were measured using an ELISA technique (Human Erythropoietin, Quantikine IVD ELISA kit, R&D Systems) and the detection limit is ∼2.5mIU/mL. The normal range is 3.1–14.9 mIU/mL. Routine blood tests (haemoglobin, kidney function and electrolytes) and eGFR calculated using the Modified Diet in Renal Disease [19] formula were performed in all patients before study enrolment.

### Office-seated and ABPM

Average seated office BP was measured after at least 5 min of rest on both arms and was calculated as the average of three consecutive measurements within a 5-min interval at baseline and during each visit at follow-up with a validated device (Omron HEM-907, Omron Healthcare Singapore PTE Ltd). The arm with the higher BP reading was used for subsequent measures. All participants underwent 24-h ABPM and heart rate monitoring using a validated device (Spacelabs 90207 or 90217 recorder; Spacelabs Healthcare, WA, USA) at baseline to exclude pseudo-resistant hypertension. At 3-month follow-up, ABPM recordings were only available from 22 patients, as some patients were participants of the Symplicity HTN-2 trial, the protocol of which was not mandatory for every patient to perform ABPM at 3-month follow-up.

### MSNA

We recorded MSNA for 22 patients to assess sympathetic activity. MSNA was recorded using microneurography in a muscle fascicle of the peroneal nerve at the fibular head as described previously [20]. Confirmation of appropriate placement of the electrode within a muscle fascicle was confirmed by a modified Valsalva maneuver, which induced a transient increase in MSNA. The nerve signal was amplified (×50,000), filtered (bandpass, 700–2000 Hz), and integrated. ECG and MSNA were digitised with a sampling frequency of 1000 Hz (PowerLab recording system, model ML785/8SP, ADInstruments). Multi-unit muscle sympathetic nerve activity was expressed as burst frequency (bursts per minute) and burst incidence (bursts per 100 heartbeats). HR was derived from continuous three-lead ECG recordings.

### Catheter-based RDN

The RDN procedure was performed as described previously [15, 16]. In brief, renal angiograms were performed via femoral access to confirm anatomic eligibility. Participants were required to have sufficiently patent renal arteries in which the catheter could be safely placed to deliver ablations. The treatment catheter (Simplicity Flex; Medtronic Ardian Inc., Palo Alto, CA) was introduced into each renal artery using a guided catheter. Visceral pain at the time of energy delivery was managed with intravenous analgesics and sedatives.

### Statistical analysis

Changes in BP, ABPM, Epo levels, and renal function at 3-month follow-up were compared with baseline measurements using a paired *t* test. Correlations were assessed by using a Pearson correlation coefficient. Data were presented as mean ± SD. A value of *p* < 0.05 was considered significant. All statistical analyses were performed with SPSS statistical software (version 19.0, SPSS Inc., Chicago, IL).

## Results

### Baseline characteristics

Baseline clinical characteristics of all patients are summarised in Table 1. The mean age of patients was 61 ± 12 years. Body mass index (BMI) was 30.9 ± 4.7 kg/m^2^. Coronary artery disease was known in 36.4%, cerebrovascular disease in 9.1% and type 2 diabetes mellitus in 27.3% of all patients. On average, patients were taking 4.9 ± 1.7 antihypertensive drugs, including angiotensin-converting enzyme inhibitors (ACEI), angiotensin II receptor blockers (ARB), β-blockers, calcium-channel blockers, diuretics, aldosterone antagonists, α-blockers and centrally acting sympatholytic agents (Table 1).

**Table 1:** Baseline characteristics of all patients

Characteristics	
Age (years)	61 ± 12
Gender, F/M	7/26
BMI (kg/m^2^)	30.9 ± 4.7
Type 2 diabetes mellitus, *n* (%)	9 (27.3)
Coronary artery disease, *n* (%)	12 (36.4)
Cerebrovascular disease, *n* (%)	3 (9.1)
Duration of hypertension (years)	19 ± 11
Office SBP (mm Hg)	165 ± 16
Office DBP (mm Hg)	85 ± 18
Office heart rate (beats/min)	62 ± 13
Medications (*n*)	4.9 ± 1.7
ACEI, n (%)	17 (51.5)
ARB, n (%)	26 (78.8)
β-blocker, *n* (%)	18 (54.5)
Calcium-channel blockers, *n* (%)	28 (84.8)
Diuretics (thiazide type or loop), *n* (%)	27 (81.8)
Aldosterone antagonists, *n* (%)	12 (36.4)
α-Blockers, *n* (%)	6 (18.2)
Centrally acting sympatholytics, *n* (%)	18 (54.5)

Data are presented as mean ± SD and percentage (%)

*BMI* body mass index, *ACEI* angiotensin-converting enzyme inhibitor, *ARB* angiotensin II receptor blocker, *SBP* systolic blood pressure, *DBP* diastolic blood pressure

### Procedure characteristics

Renal angiograms were performed before the introduction of the RF treatment catheter via femoral access for determination of anatomic eligibility, and absence of significant vascular pathology was confirmed in all patients. An average of 9.9 ± 2.5 ablation treatments were delivered in each patient per renal artery. Angiographic evaluation before and directly after RDN revealed no compromise of the treated arteries. There were no intra- or periprocedural complications. No short-term (at 3-month follow-up) adverse events related to the procedure were noted in any of the treated patients.

### Effects of renal denervation on plasma Epo level and serum biochemistry

**Fig. 1:**
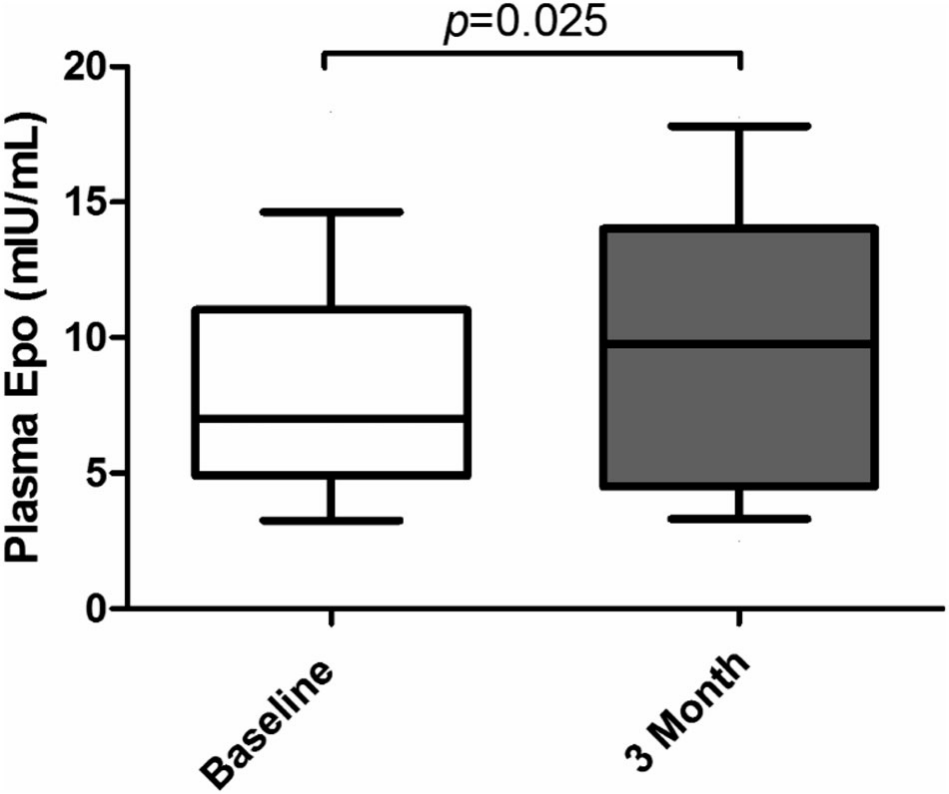
Plasma levels of Epo before and 3 months after RDN

Plasma levels of Epo were significantly higher at the 3-month follow-up compared to baseline for all patients (7.81 ± 3.68 versus 9.88 ± 5.06 mIU/mL; *P* = 0.025) (Fig. 1). Haemoglobin slightly increased by 1.29 ± 7.60 g/L after 3 months, but this was not significant (*p* = 0.352). There were no changes in red blood cell count, haematocrit, MCV, MCH, MCHC and RDW after RDN. No significant alterations were found in kidney function assessed by eGFR and serum creatinine (76.24 ± 12.35 versus 76.21 ± 14.36 ml/min per 1.73 m^2^, *p* = 0.986; 83.48 ± 16.37 versus 84.33 ± 18.48 umol/L, *p* = 0.679), respectively (Table 2). The changes in levels of serum potassium (4.03 ± 0.35 versus 4.09 ± 0.40 mmol/L; *p* = 0.377) and sodium (139 ± 3 versus 139 ± 2 mmol/L; *p* = 0.270) were also unchanged after RDN.

**Table 2:** Changes in Epo, haemoglobin, blood pressure, MSNA and renal function after RDN at 3 months follow-up

Parameter	Baseline	3 Month	Change	*p* Value
Plasma erythropoietin (mIU/mL)	7.81 ± 3.68	9.88 ± 5.06	2.07 ± 5.07	0.025
eGFR (mL/min/1.73 m^2^)	76.24 ± 12.35	76.21 ± 14.36	−0.03 ± 10.09	0.986
Serum creatinine (umol/L)	83.48 ± 16.37	84.33 ± 18.48	0.85 ± 11.69	0.679
Haemoglobin (g/L)	141 ± 10	142 ± 10	1.28 ± 6.51	0.274
MSNA (bursts/min)	47 ± 13	45 ± 13	−2 ± 12	0.432
MSNA (bursts/100 heartbeats)	76 ± 19	71 ± 17	−5 ± 17	0.195
Office SBP (mmHg)	165 ± 16	150 ± 19	−15 ± 19	<0.001
Office DBP (mmHg)	85 ± 17	80 ± 16	−5 ± 12	0.033
Office PP (mmHg)	80 ± 20	72 ± 20	−8 ± 18	0.014
ABPM (*n* = 22)				
Mean SBP (mmHg)	145 ± 16	140 ± 18	−5 ± 11	0.036
Mean DBP (mmHg)	80 ± 11	77 ± 12	−3 ± 7	0.024
Daytime SBP (mmHg)	149 ± 16	143 ± 19	−6 ± 12	0.039
Daytime DBP (mmHg)	83 ± 11	80 ± 13	−3 ± 7	0.026
Night-time SBP (mmHg)	138 ± 18	135 ± 18	−3 ± 13	0.323
Night-time DBP (mmHg)	75 ± 12	71 ± 12	−4 ± 9	0.059
Heart rate (beats/min)	63 ± 10	64 ± 10	1 ± 5	0.410

Data are presented as mean ± SD

*eGFR* estimated glomerular filtration rate, *MSNA* muscle sympathetic nerve activity, *ABPM* ambulatory blood pressure monitoring, *SBP* systolic blood pressure, *DBP* diastolic blood pressure

**Fig. 2:**
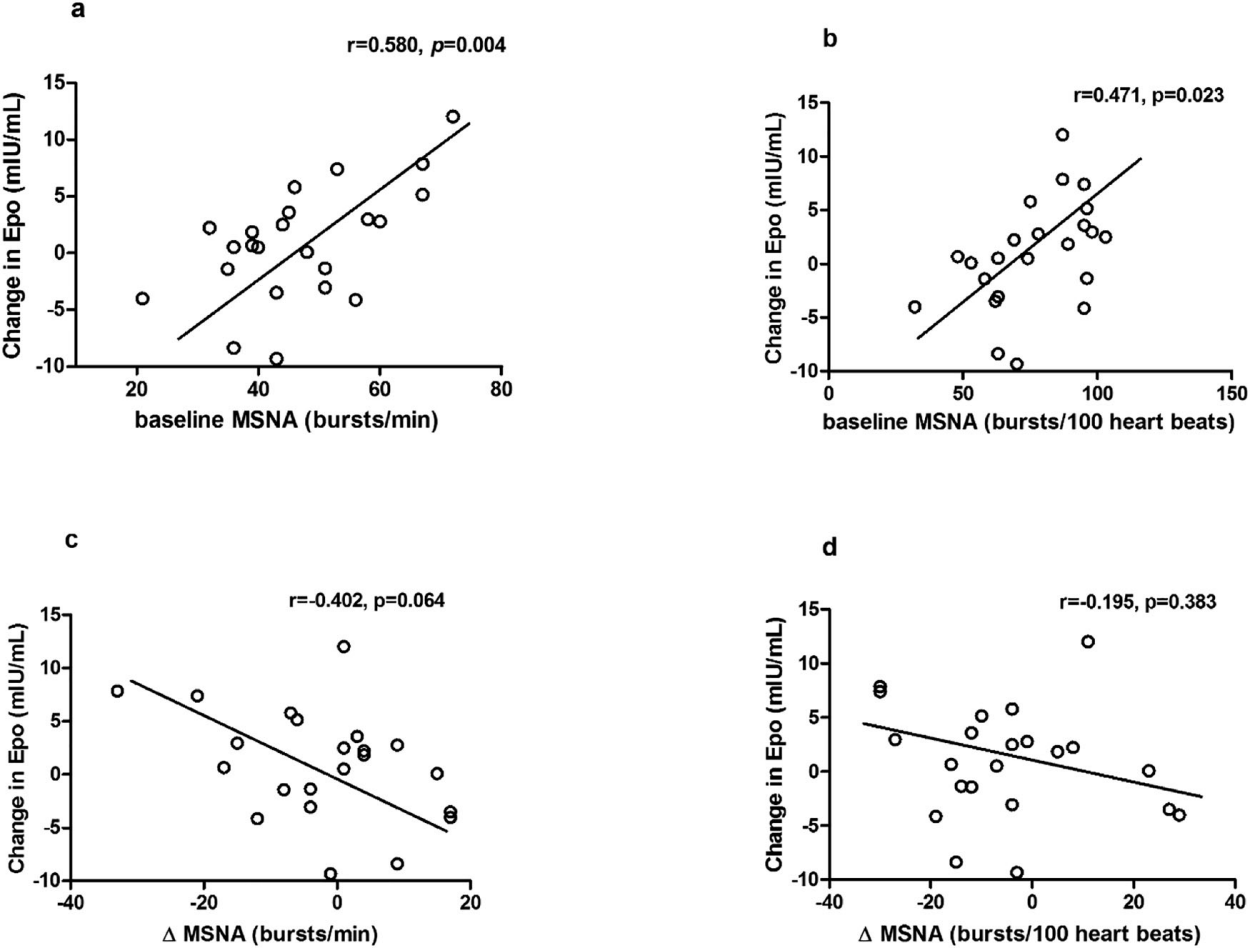
The relationship between changes in Epo and baseline MSNA (**a**, **b**). The correlation of changes in Epo and changes in MSNA (**c**, **d**). MSNA, muscle sympathetic nerve activity

Univariate analysis confirmed a significant relationship between RDN-related changes of Epo level and burst frequency of baseline MSNA (*r* = 0.580, *p* = 0.004) and burst incidence of baseline MSNA (*r* = 0.471, *p* = 0.023). The RDN-induced changes in burst frequency of MSNA tended to correlate inversely with changes in Epo levels (*r* = −0.402; *p* = 0.064). This, however, did not reach statistical significance. Similarly, there was no significant correlation between post-RDN changes in Epo levels and burst incidence of MSNA (*r* = −0.195, *p* = 0.383). (Fig. 2) Similarly, there was no difference in the change of Epo 3 months following RDN between patients on or off beta blockers at baseline (Beta-Blocker: 1.83 versus no Beta-Blocker: 2.36, *p* = 0.12).

### Effects on blood pressure

At baseline, overall mean sitting office SBP was 165 ± 16 mmHg and mean seated office DBP was 85 ± 17 mmHg, with a heart rate of 62 ± 13 bpm. At the 3-month follow-up, average office SBP, DBP and pulse pressure (PP) were significantly reduced by 15 ± 19, 5 ± 12 and 8 ± 18 mmHg in all patients (*p* < 0.001 for SBP, *p* = 0.033 for DBP and *p* = 0.014 for PP), respectively (Fig. 4).

Analysis of ABPM revealed that RDN resulted in a significant reduction in 24 h mean SBP (145 ± 16 versus 140 ± 18 mm Hg; *p* = 0.036) and 24 h mean DBP (80 ± 11 versus 77 ± 12 mmHg; *P* = 0.024).  Changes in day-time SBP (149 ± 16 versus 143 ± 19 mmHg; *P* = 0.039) and day-time DBP (83 ± 11 versus 80 ± 13 mm Hg; *P* = 0.026) from baseline were also statistically significant (Fig. 4).

**Fig. 3:**
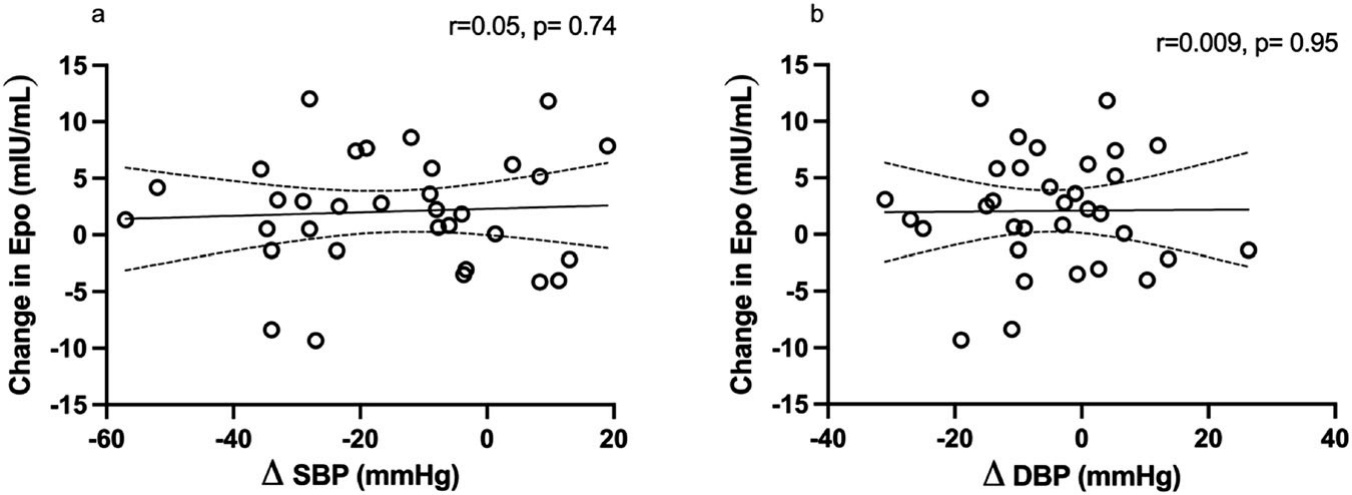
The linear relationships between the change in serum Epo concentration (mIU/mL) and systolic blood pressure reduction at 3 months (**a**) and diastolic blood pressure reduction (**b**)

**Fig. 4:**
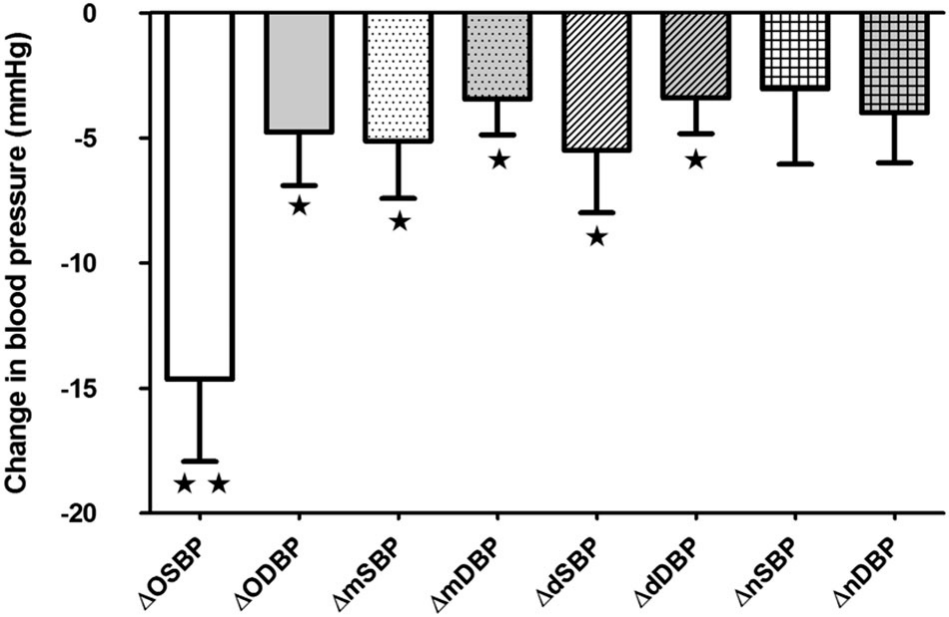
Changes in BP assessed by office BP and ABPM (mean ± SEM). OSBP office systolic blood pressure, ODBP office diastolic blood pressure, mSBP mean systolic blood pressure of ABPM, mDBP mean diastolic blood pressure of ABPM, dSBP day-time systolic blood pressure of ABPM, dDBP day-time diastolic blood pressure of ABPM, nSBP night-time systolic blood pressure of ABPM, nDBP night-time diastolic blood pressure of ABPM, **p < 0.01; *p < 0.05

In contrast to effects of RDN on 24 h mean BP and daytime BP of ABPM, RDN demonstrated a less pronounced reduction in night-time BP of ABPM, which did not reach the level of statistical significance. Three months after RDN, night-time SBP and DBP were reduced from 138 ± 18 to 135 ± 18 mmHg (*p* = 0.323) and from 75 ± 12 to 71 ± 12 mmHg (*p* = 0.059), respectively (Table 2 and Fig. 4).

We did not find any statistically significant correlation between RDN-related changes of plasma Epo and RDN-related changes of SBP or DBP levels (Fig. 3), neither for office SBP (*r* = 0.058, *p* = 0.748) nor for 24 h mean SBP of ABPM (*r* = −0.089, *p* = 0.695). Likewise, we did not detect a significant correlation between MSNA burst frequency and systolic blood pressure change at 3 months (*p* = 0.29, *r* = 0.24) with similar findings for burst incidence and changes in systolic blood pressure (*p* = 0.07, *r* = 0.4).

## Discussion

The present study was performed to elucidate whether catheter-based RDN impacts the renal synthesis of Epo, as has been previously reported in animal models. Above all, our findings provided the first human evidence that plasma levels of Epo were substantially increased in patients with resistant hypertension at 3 months after renal nerve ablation in the absence of changes in haemoglobin, while significantly reducing blood pressure. Second, the elevated EPO level was still within the normal physiological range (from 7.81 ± 3.68 to 9.88 ± 5.06 mIU/mL), which indicates that RDN does not excessively increase the production of Epo within 3 months post-procedure. Third, changes in Epo levels correlated with baseline MSNA, such that the increase in Epo was most pronounced in patients with high baseline MSNA. The RDN-induced changes in MSNA tended to correlate inversely with changes in Epo levels. These findings suggest that the RDN-induced increase in Epo levels may be mediated via a reduction in sympathetic nerve activity either globally or downstream of renal sympathetic innervation.

Although the structure and function of Epo is well documented, apart from classical pathways of regulating renal Epo during hypoxia and anaemia, alternative pathways in regulation of the renal synthesis of Epo are still poorly understood [3, 21]. The kidney has rich innervation, which accompanies intrarenal blood vessels, and the sympathetic renal nerves are distributed to all segments of the intrarenal vasculature in the renal cortex [22]. The peritubular fibroblasts in the renal cortex are the main production site of Epo after birth. Since the 1960s, multiple studies have investigated the role of renal nerves on Epo synthesis in a variety of RDN animal models, however, the published data in relation to changes in Epo synthesis are conflicting.

RDN has emerged as a novel treatment option for patients with resistant hypertension. The above-mentioned inconsistent results prompted us to investigate the influence of renal synthesis of Epo after RDN in humans. In the present study, we found that circulating Epo levels were increased after RDN. This is the first human data confirming changes in Epo levels after RDN and occurs in the absence of significant respiratory pathology causing hypoxia. The precise mechanism of how this occurs is currently unclear and will require concerted research effort to determine. We found our results to be consistent with most RDN animal studies [5, 10, 11]. Fink et al. found that Epo levels were markedly reduced by bilateral RDN in 5 h, but after 18 h of hypoxia, plasma Epo levels in renal denervated rabbits did not differ significantly from those of sham-operated controls [12]. The experimental observation time was too short, which may have been the possible reason for the difference in results. Other investigations, however, [13] have indicated RDN had no effect on Epo production by hypoxic stimuli in rats. Indeed, hypoxia is known to be a reliable stimulus to increase Epo and is the principal driver of Epo production. Indeed, the classical example in humans is that of chronic obstructive pulmonary disease in which chronic hypoxaemia drives increased Epo release [23], which can progress to polycythaemia [24].

Our findings corroborate with two previous studies of spinal cord injury where renal afferent and efferent nerves were non-functional, demonstrating  increased Epo levels [25, 26]. Serum Epo levels have also been reported to be slightly elevated after renal transplantation [27], a scenario where all renal nerves are severed. Although in different clinical settings, the contribution of the endogenous and extrarenal sites to changes in Epo levels cannot be excluded with certainty, our present investigation clearly supports the ability of denervated kidneys to increase Epo production appropriately.

The control of Epo production is not purely downstream of local hypoxic signalling in the kidney. Raised intracranial pressure and medullary, but not renal, hypoxia are also stimulators of renal Epo production [21]. Whilst intraneural Epo is inducible by cerebral hypoxia [28]—with some distinct neuroplastic effects [29]—the elevated serum Epo is abolished following nephrectomy, implying some form of neural or neurohumoral factors which drive this process. Indeed, this appears to be under some degree of sympathetic control, with hypoxia-driven Epo release reduced in beta-adrenergic receptor knockout mice, mirrored by littermates who underwent adrenalectomy [30]. Beta-blockade alone appears sufficient to reduce Epo production following a hypoxic bout [31]. Interestingly, we found no difference in change in Epo between patients prescribed beta blockers and those without beta blockers at 3 months of follow-up. Fibroblasts producing Epo are known to express the AT1 receptor, which binds angiotensin II [32], and administration of Angiotensin II leads to renal EPO production blocked by losartan [33]. This occurs even in normoxic conditions. Fundamentally, these results imply that both direct and indirect sympathetic activation at kidney level can alter (increase) Epo synthesis, whereas  our findings imply sympathoinhibition activates Epo-producing mechanisms. Our work shows correlations between Epo changes at 3 months and baseline MSNA. MSNA itself does correlate with renal noradrenaline spillover [34], and while an indirect measure of renal sympathetic nerve activity—our findings indeed suggest that the RDN-induced inhibition  of elevated renal sympathetic drive may be responsible for this finding. Another relevant consideration is the time scale of available investigations. Most experimental work in this setting has been performed acutely with protocols in the magnitude of hours to days as opposed to months, as we provide here in our human study. Longer-term data in both animals and humans may be useful to better define the role of sympatho-inhibition on Epo production.

Epo is an essential component for the maturation of erythrocytes, with dysregulation of this system leading either to normocytic anaemia or polycythaemia [3]. Our work demonstrates a modest increase in Epo following RDN at 3 months—the plasma Epo level increased by 2.07 ± 5.07 mIU/mL, remaining clearly within the physiological range. This may explain the recent observations of mild increases in haemoglobin following RDN in CKD [17].

One cannot ignore the evidence that hypertension develops or worsens in 20–30% of renal patients treated with recombinant human erythropoietin (r-HuEPO) [35], and two large clinical trials indicated that r-HuEPO treatment might be associated with an increase in cardiovascular events in patients with chronic kidney disease [36, 37]. However, lower doses of r-HuEPO (~3000 IU) have been reported to show no immediate hypertensive effects in humans [38]. The effects of endogenously elevated Epo  may  differ from those evoked by exogenous administration. The populations studies in the above mentioned trials were patients with renal failure, and as such, elevating haemoglobin was the main goal of the treatment. An increase in cardiovascular events, including vascular access thrombosis, stroke, and myocardial infarction, has been associated with a rapid increase in haemoglobin [39]. In the present study, while haemoglobin was numerically increased  after RDN, this did not reach statistical significance. There was also no correlation between changes in Epo and changes in SBP. While there is the possibility that this increase in Epo post-RDN may somewhat diminish the systolic blood pressure reduction, this does not appear—at least in this study—to be of a magnitude sufficient to undermine the broader antihypertensive effect of the procedure. Similarly, there is a lack of direct correlation between MSNA parameters and the systolic blood pressure response. Given that MSNA recordings were available in only a subset of our participants, this may be an issue of insufficient statistical power. A clear trend for a correlation between burst incidence and systolic blood pressure was evident.

In the current study, we found that changes in plasma Epo levels after RDN were related to the baseline MSNA level. The changes in Epo levels trended to be inversely relation to changes in MSNA. That means hypertensive patients who had higher sympathetic activity before RDN may increase Epo production following RDN more than participants with lower resting MSNA. It would therefore appear possible that the reduction of sympathetic drive to the kidney helps to mediate this response. These results indicate that the reduction in sympathetic activation may mediate the increase in Epo levels after RDN. This contrasts with the theory that renal sympathetic activation induces Epo formation. Interestingly, in many studies, increased renal sympathetic nerve activity is a potent stimulus for Epo formation [40–42], and β-blockers via antagonism of the renal β receptors can reduce Epo secretion under hypoxic conditions [12, 31, 43]. It is worth noting that this Epo increase is unlikely to be related to changes in direct renal haemodynamics. It is well-established in humans that renal blood flow does not significantly reduce following renal denervation in the 3-month post-procedure [44]. This is further reflected in the lack of eGFR fall following renal denervation in either the short term [45] or the long term [46]. As such, it is unlikely that the increased Epo is a reflection of renal hypoxia or reduced renal blood flow.

In our study, RDN reduced office SBP by 15 ± 19 mmHg, which is slightly less than the results noted in previous studies [14–16, 47]. Higher baseline SBP remains the most significant independent predictor of SBP response [15]. The patients enrolled in the current study have lower baseline office SBP (165 ± 16 mmHg), while the patients in Symplicity HTN-2 have higher baseline SBP (178 ± 23 mmHg), which may explain the differences.

Consistent with the effects of RDN on office BP, RDN demonstrated pronounced reduction in mean BP and daytime BP assessed by means of ABPM. Three months after RDN, mean SBP and day-time SBP were reduced by 5 and 7 mmHg, respectively. The fall in night-time BP was not  statistically significant. In general, humans tend to have higher sympathetic outflow in the day than at night. This may account for the more pronounced decline of daytime blood pressure compared to nighttime after RDN (Fig. 4).

### Limitations

There are several limitations of our study that need to be recognised. First, the study design was observational, non-randomised, with no control group. Second, the sample size of our study was small but sufficiently large to detect effects on the changes in Epo levels and BP. Third, short-term follow-up of 3 months as reported here may not be sufficient to explore the full extent of the effects of RDN on Epo level and the related changes in BP. It is now well understood that the effects of RDN continue to compound out to 3 years and are stable out to the long term [48]. This needs to be explored in further, longer-term follow-up studies. Furthermore, our study does not provide enough mechanistic explanations of our findings; further research is strongly encouraged.

## Conclusion

In humans under normoxic conditions, renal denervation appears to increase Epo levels within the physiological range. This was insufficient in our cohort to cause a statistically or clinically significant increase in serum haemoglobin concentration. The change in Epo at 3-month follow-up appears to correlate with resting, pre-procedural sympathetic drive as measured by MSNA. Further work will be required to explore the mechanistic underpinnings of these findings.
